# Loss of hepatic *Flcn* protects against fibrosis and inflammation by activating autophagy pathways

**DOI:** 10.1038/s41598-021-99958-7

**Published:** 2021-10-28

**Authors:** Mathieu Paquette, Ming Yan, Josué M. J. Ramírez-Reyes, Leeanna El-Houjeiri, Marco Biondini, Catherine R. Dufour, Hyeonju Jeong, Alain Pacis, Vincent Giguère, Jennifer L. Estall, Peter M. Siegel, Étienne Audet-Walsh, Arnim Pause

**Affiliations:** 1 Goodman Cancer Research Center, McGill University, Montreal, QC Canada; 2 Department of Biochemistry, McGill University, Montréal, QC Canada; 3 Canadian Centre for Computational Genomics, McGill Genome Centre, Montréal, QC Canada; 4 Endocrinology - Nephrology Research Axis, Centre de Recherche du CHU de Québec - Université Laval, Québec, QC Canada; 5 Institut de Recherches Cliniques de Montréal (IRCM), Montréal, QC Canada; 6 Department of Medicine, McGill University, Montréal, QC Canada

**Keywords:** Liver diseases, Transcription factors

## Abstract

Non-alcoholic fatty liver disease (NAFLD) is the most frequent liver disease worldwide and can progress to non-alcoholic steatohepatitis (NASH), which is characterized by triglyceride accumulation, inflammation, and fibrosis. No pharmacological agents are currently approved to treat these conditions, but it is clear now that modulation of lipid synthesis and autophagy are key biological mechanisms that could help reduce or prevent these liver diseases. The folliculin (FLCN) protein has been recently identified as a central regulatory node governing whole body energy homeostasis, and we hypothesized that FLCN regulates highly metabolic tissues like the liver. We thus generated a liver specific *Flcn* knockout mouse model to study its role in liver disease progression. Using the methionine- and choline-deficient diet to mimic liver fibrosis, we demonstrate that loss of *Flcn* reduced triglyceride accumulation, fibrosis, and inflammation in mice. In this aggressive liver disease setting, loss of *Flcn* led to activation of transcription factors TFEB and TFE3 to promote autophagy, promoting the degradation of intracellular lipid stores, ultimately resulting in reduced hepatocellular damage and inflammation. Hence, the activity of FLCN could be a promising target for small molecule drugs to treat liver fibrosis by specifically activating autophagy. Collectively, these results show an unexpected role for *Flcn* in fatty liver disease progression and highlight new potential treatment strategies.

## Introduction

Non-alcoholic fatty liver disease (NAFLD) is a disease spectrum characterized by triglyceride accumulation in hepatocytes and is associated with obesity, insulin resistance, type 2 diabetes, and dyslipidemia^1^. Initially exhibiting NAFLD, approximately one-third of patients will progress toward non-alcoholic steatohepatitis (NASH)^2^. Furthermore, approximately one-third of patients with NASH will progress to cirrhosis and final liver dysfunction^3,4^. There is currently no pharmacological agent approved to treat NASH or to slow its progression^5,6^. The only proven treatments are weight loss and increased physical activity, with fibrosis improvement in only 10–20% of patients^5,6^.

NASH is characterized by hepatocellular injury, immune cell-mediated inflammation, and progressive liver fibrosis^7^. The accumulation of lipid intermediates within hepatocytes causes lipotoxicity, cellular stress, and eventually cell death via distinct mechanisms^7^. Fatty acid modulates cell signaling, disrupts metabolic homeostasis, and induces ER stress^8,9^. Chronic liver injury eventually leads to fibrosis as a consequence of chronic wound-healing responses initiated by dying hepatocytes^10^. In a healthy state, wound-healing restores liver function and structure by recruiting liver progenitor populations and inflammatory cells, which can release angiogenic and matrix remodeling factors^11^. In NASH, wound-healing is perpetual, and the persistent inflammation prevents the termination of the process thus perpetuating fibrogenesis and inflammation^10^. Therefore, regulation of the lipid content is crucial to maintain liver homeostasis.

AMP-activated protein kinase (AMPK) is a master regulator of cellular energy metabolism and key transducer of metabolic inputs that can stimulate the switch from lipogenesis, an anabolic ATP-consuming process, to the catabolism of fatty acids and generation of energy^12^. In the recent years, a causal role of the mechanistic target of rapamycin (mTOR) signaling pathway has also been linked to hepatic steatosis^13,14^. mTOR is a serine/threonine kinase found in two distinct complexes, mTORC1 and mTORC2, that controls various biosynthetic pathways^15^. These include autophagy and lipid metabolism, two biological pathways that are known to be tightly linked to the transition from a healthy liver to NAFLD and NASH^7,15,16^. Consequently, there was hope that inhibition of mTOR with rapalogs such as rapamycin could reduce steatosis, notably by restoring autophagy^13,14^. However, more recent studies indicate that long term treatment with these inhibitors induce liver damage and enhance tumorigenesis^17,18^. In that settings, we searched for a new therapeutic target linked to the AMPK and mTOR signaling pathways but for which its modulation would not cause long-term side effects such as rapalogs.

Folliculin (*Flcn*) loss of function has been studied in nematodes and various tissues of knockout mice, showing that *Flcn* loss leads to a chronic activation of AMPK with surprisingly beneficial effects^19–23^. For example, mice with *Flcn* knockout specifically in skeletal muscle developed a pronounced metabolic shift towards oxidative phosphorylation, increased mitochondrial biogenesis, and red colored muscles^21^. Similarly, mice with conditional *Flcn* knockout in adipocytes developed resistance to high-fat diet (HFD)-induced obesity as well as browning of white adipocytes and increased mitochondrial activity in brown fat^22,23^. In addition, FLCN is also a crucial mTORC1 regulator^24,25^. FLCN is a GTPase activating protein (GAP) toward the Rags GTPase C and D, two important proteins required for mTORC1 localization on the lysosomes. Therefore, *Flcn* loss prevents the activation of mTORC1 on the lysosomal membrane and activates autophagy, without affecting the mRNA translation machinery^23,26^. Still, little is known about how FLCN regulates a highly metabolic tissue such as the liver and, more specifically, the potential role of FLCN in liver fibrosis progression has never been investigated.

In this study, we generated a liver specific *Flcn* knockout mouse model challenged with a methionine/choline deficient, high fat, high sucrose (MCD) diet, mimicking transition to liver fibrosis in vivo. We demonstrate that liver-*Flcn* KO mice were protected from MCD-induced triglyceride accumulation and liver damage. Importantly, loss of *Flcn* in liver tissue resulted in increased autophagic activity and nuclear localization of TFE3 and TFEB, reduced fibrosis, and reduced inflammation, underlying potential mechanisms for liver fibrosis protection. Altogether, these results identified FLCN as a novel potential therapeutic target for NAFLD and NASH.

## Results

### Hepatic loss of *Flcn* protects against high fat diet stimulated NAFLD

**Figure 1:**
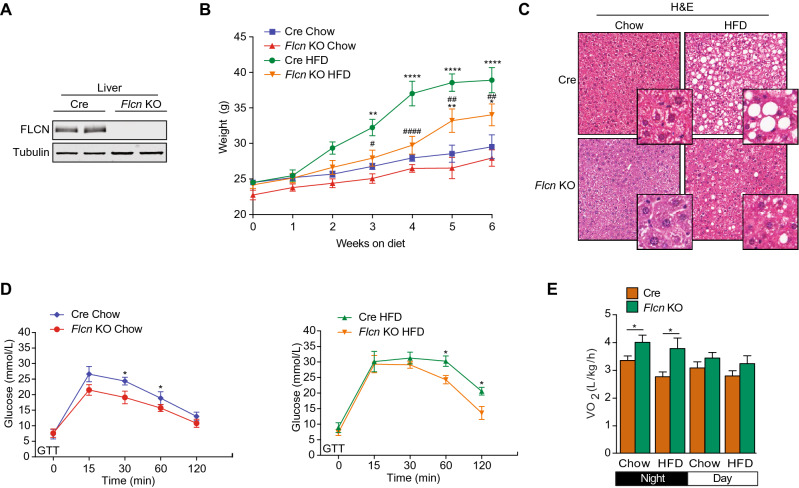
Hepatic loss of *Flcn* protects against high fat diet stimulated NAFLD. (**A**) Western blot detection of hepatic FLCN protein levels from 3-month-old mice fed standard chow. Full-length blots are presented in Supplementary Fig. 5. (**B**) Mouse body weight when fed either on chow or high fat diet (HFD) over a period of 6 weeks (mean ± SEM, two-way ANOVA, *P < 0.05; **P < 0.01; ****P < 0.0001 compared to Cre-chow, ^#^P < 0.05; ^##^P < 0.01; ^####^P < 0.0001 compared to Cre-HFD; n = 6 mice per condition). (**C**) H&E stainings in liver sections of mice fed either chow or high-fat diet (HFD) over a period of 6 weeks. Images are representative of 6 mice per condition. (**D**) Blood glucose during a GTT in chow or HFD-fed mice following a 16-h fast and intraperitoneal glucose administration of 2 g per kg of body weight (mean ± SEM, two-way ANOVA, *P < 0.05; ***P < 0.001; n = 6 mice per condition). (**E**) Quantification of metabolic cage analysis of mice fed chow or a HFD for 2 months. Circadian VO_2_ consumption levels during a 12 h light: 12 h dark cycle (mean ± SEM, two-way ANOVA, **P < 0.01; ****P < 0.0001; n = 6 mice per condition).

To study the role of FLCN in the liver, we generated a liver-specific *Flcn* KO (liver-*Flcn*^−/−^) mouse model by crossing *Flcn*^lox/lox^ mice with albumin-cre^+/−^ mice (Fig. 1A and supplemental Fig. 1A,B)^22^. Liver-*Flcn*^−/−^ mice were born at the expected Mendelian frequency, displayed no developmental defects, survived without difficulty, were fertile, and had similar weights compared with wild-type mice at weaning (4 weeks of age).

We initially established whether *Flcn* loss in the liver affects whole-body metabolism by feeding them either normal chow or a high fat diet (HFD) containing 60% kcal from fat for 6 weeks. Control mice (Cre mice) gained significantly more weight than liver-*Flcn*^−/−^ mice (~ 50% vs ~ 20%, respectively) on a HFD challenge (Fig. 1B). Hematoxylin and eosin (H&E) staining revealed a strong reduction in fat droplets in livers of *Flcn* KO mice when challenged with HFD compared to Cre mice (Fig. 1C). To understand how liver-specific loss of *Flcn* protected against HFD-induced obesity, we then examined if it improved systemic energy metabolism. First, glucose and insulin tolerance tests showed that loss of *Flcn* in hepatocytes significantly increased glucose uptake and enhanced insulin responsiveness, respectively, under both chow and a HFD regimen (Fig. 1D and supplemental Fig. 1C). Secondly, we employed the use of metabolic cages capable of simultaneously measuring animal food intake, physical activity and energy expenditure. Mice fed either chow or HFD were examined individually in metabolic cages. Liver-*Flcn*^−/−^ mice were found to be more active during the dark phase of the circadian cycle concomitant with increased maximal oxygen consumption (VO_2_) compared to Cre controls independent of diet (Fig. 1E and supplemental Fig. 1D). Together, the data suggest that the increased energy expenditure and metabolic activity of liver-*Flcn*^−/−^ mice are underlying factors in the resistance of these animals to HFD-induced obesity. Hence, loss of *Flcn* specifically in the liver improved metabolic homeostasis and prevented weight gain in a diet-induced obesity model.

### *Flcn* loss protects against MCD diet-induced fibrosis and inflammation

Since liver phenotypes associated with a HFD are highly associated with obesity and whole-body insulin resistance, the effects observed in this experimental setting may have been an indirect consequence of reduced adiposity. To test whether protection against steatosis and NAFLD was due to cell-autonomous effects of *Flcn* KO in hepatocytes, we investigated whether loss of hepatic *Flcn* was also protective in a model of liver fibrosis that does not cause weight gain or insulin resistance. To this end, we subjected Cre and liver-*Flcn*^−/−^ mice to an MCD diet for six weeks. The MCD diet is a classical dietary model of liver fibrosis. Although the diet comprises high sucrose (46%) and fat (10%), it lacks methionine and choline, which are indispensable for hepatic mitochondrial β-oxidation and very low-density lipoprotein (VLDL) synthesis. This diet is a model of a metabolic challenge and results in a significant and rapid onset of a NASH-like phenotype with fibrosis, inflammation, oxidative stress, and liver cell death^27^. As previously described^28^, we show that this diet induced significant weight loss (Supplemental Fig. 2A).

**Figure 2:**
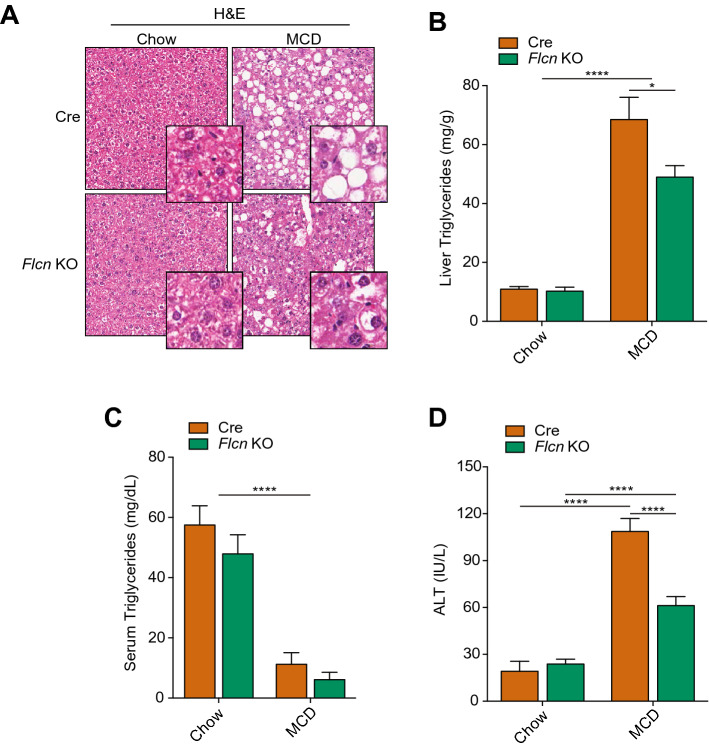
*Flcn* loss reduces MCD diet-induced liver triglyceride accumulation and liver damage. (**A**) H&E staining in liver sections of mice fed either chow or methionine/choline deficient diet (MCD) over a period of 6 weeks. Images are representative of 8 mice per condition. (**B**) Liver triglycerides quantification in mice fed as described in (**A**) (mean ± SEM, two-way ANOVA, *P < 0.05; **P < 0.01; ****P < 0.0001; n = 8 mice per condition). (**C**) Serum triglycerides quantification in mice fed as described in (**A**) (mean ± SEM, two-way ANOVA, *P < 0.05; ****P < 0.0001; n = 8 mice per condition). (**D**) Alanine transaminase (ALT) quantification in mice fed as described in (**A**) (mean ± SEM, two-way ANOVA, **P < 0.01; ****P < 0.0001; n = 8 mice per condition).

As seen for mice under HFD (Fig. 1), hepatic loss of *Flcn* protected against hepatic lipid accumulation in mice under the MCD diet, as shown with the H&E staining (Fig. 2A). Indeed, specific quantification of the total triglycerides in the liver tissue also showed a significant reduction in fat accumulation in *Flcn* KO livers (Fig. 2B). There was no significant difference in serum triglycerides, serum total cholesterol, serum free cholesterol, and serum cholesterol esters between Cre and liver-*Flcn*^−/−^ mice (Fig. 2C and Supplemental Fig. 2B–D). These results suggest that liver-specific loss of *Flcn* reduces fat accumulation not through increased fat export into the blood. Levels of alanine transaminase (ALT), a hepatic enzyme released following liver damage due to hepatocyte death, increased significantly in Cre mice under MCD diet, an effect blunted in liver-*Flcn*^−/−^ mice, thus supporting their protection against liver damage (Fig. 2D).

**Figure 3:**
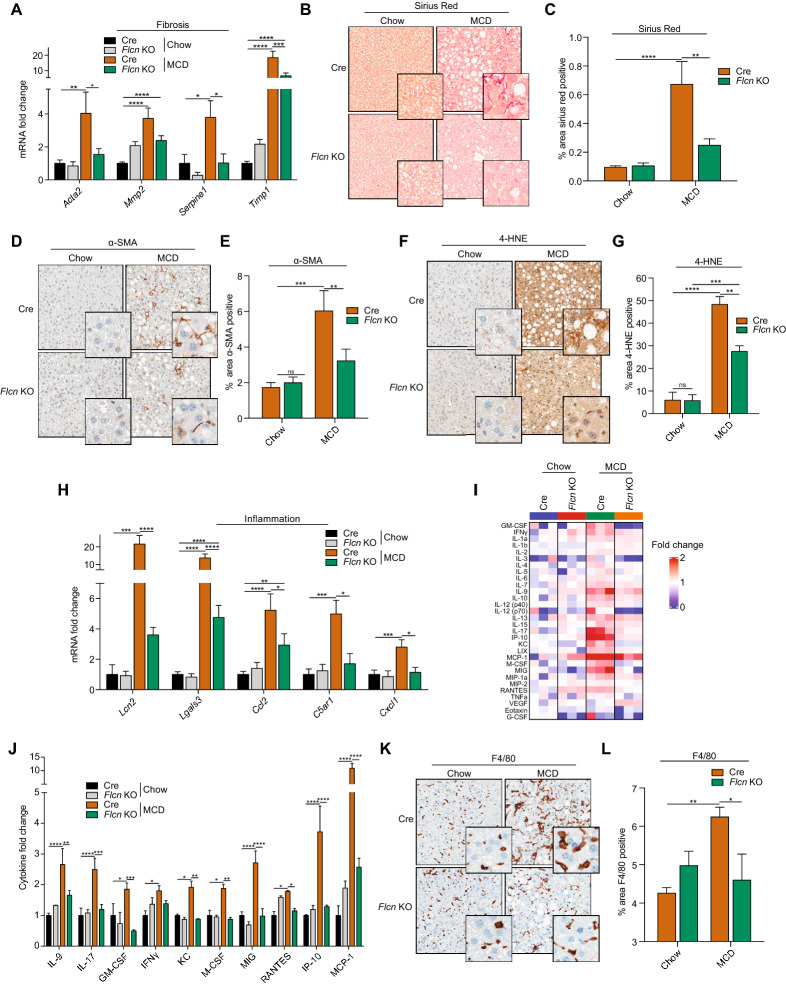
*Flcn* loss reduces MCD diet-induced fibrosis and inflammation. (**A**) Relative quantitative real‐time PCR analysis of fibrosis-related genes mRNA transcript levels in livers of mice fed either on chow or methionine/choline deficient diet (MCD) over a period of 6 weeks (mean ± SEM of the RNA fold change of indicated mRNAs; two-way ANOVA, *P < 0.05, **P < 0.01, ***P < 0.001, ****P < 0.0001; n = 8 mice per condition). (**B**) Sirius red staining in liver sections of mice fed as described in (**A**). Images are representative of 6 mice per condition. (**C**) Quantification of the sirius staining in liver sections of mice fed as described in (**A**) (mean ± SEM, two-way ANOVA, **P < 0.01; ****P < 0.0001; n = 6 mice per condition). (**D**) α-SMA Immunohistochemistry (IHC) staining in liver sections of mice fed as described in (**A**). Data are representative of 4 mice per condition. (**E**) Quantification of α-SMA IHC staining in liver sections of mice fed as described in (**A**) (mean ± SEM, two-way ANOVA, **P < 0.01; ***P < 0.001; n = 4 mice per condition). (**F**) 4-HNE Immunohistochemistry (IHC) staining in liver sections of mice fed as described in (**A**). Data are representative of 4 mice per condition. (**G**) Quantification of 4-HNE IHC staining in liver sections of mice fed as described in (**A**) (mean ± SEM, two-way ANOVA, **P < 0.01; ***P < 0.001, ****p < 0.0001; n = 4 mice per condition). (**H**) Relative quantitative real‐time PCR analysis of inflammation-related genes mRNA transcript levels in livers of mice fed as described in (**A**) (mean ± SEM of the RNA fold change of indicated mRNAs; two-way ANOVA, *P < 0.05, **P < 0.01, ****P < 0.0001; n = 8 mice per condition). (**I-J**) Mouse cytokines and chemokines quantification in liver homogenates of mice fed as described in (**A**) (mean ± SEM, two-way ANOVA, *P < 0.05, **P < 0.01, ***P < 0.001, ****P < 0.0001; n = 3 mice per condition). (**K**) F4/80 Immunohistochemistry (IHC) staining in liver sections of mice fed as described in (**A**). Data are representative of 4 mice per condition. (**L**) Quantification of F4/80 IHC staining in liver sections of mice fed as described in (**A**) (mean ± SEM, two-way ANOVA, *P < 0.05; **P < 0.01; n = 4 mice per condition).

One hallmark of NASH is fibrosis, which generates liver damage and eventually leads to hepatocyte death and liver dysfunction^7^. In our mouse model, relative mRNA transcript levels of genes related to fibrosis were induced in Cre mice fed the MCD diet, but generally to a lower extent in liver-*Flcn*^−/−^ mice (Fig. 3A). Sirius Red staining also revealed that fibrosis was aggravated in Cre mice challenged on the MCD diet, but this effect was diminished in liver-*Flcn*^−/−^ mice (Fig. 3B, quantified in C). Immunohistochemistry staining of ⍺-smooth muscle actin (⍺-SMA), a fibrosis-related marker, was significantly increased in Cre mice challenged with the MCD diet and again loss of *Flcn* was protective (Fig. 3D, quantified in E). Finally, immunohistochemistry staining of 4-Hydroxynonenal (4-HNE), a lipid peroxidation marker, was significantly increased in Cre mice challenged with the MCD diet but diminished in liver-*Flcn*^−/−^ mice (Fig. 3F, quantified in G). Taken together, these results demonstrate that liver-specific loss of *Flcn* protects against fibrosis.

NASH development is conjointly characterized by increased inflammation that contributes to liver damage and disease progression. In our mouse model, relative mRNA transcript levels of genes involved in inflammation were lower in liver-*Flcn*^−/−^ mice compared to Cre mice when fed the MCD diet (Fig. 3H). Using a mouse protein cytokine array, we determined the cytokine and chemokine secretion profiles in mouse livers. Cytokine protein levels were higher in Cre mice challenged with the MCD diet compared to liver-*Flcn*^−/−^ mice (Fig. 3I,J). More specifically, some macrophage chemo-attractants (RANTES, MCP-1, IP-10) were induced in Cre mice fed the MCD diet but to a lesser extent in liver-*Flcn*^−/−^ mice (Fig. 3J). Normal livers are infiltrated by specialized macrophages that contribute to inflammation when activated. Notably, immunohistochemistry staining of F4/80, a marker of murine macrophages, was increased in Cre mice fed the MCD diet, but were not increased in liver-*Flcn*^−/−^ mice (Fig. 3K, quantified in L). Collectively, our data indicates that liver-specific loss of *Flcn* reduces fibrosis and inflammation in mice challenged with the MCD diet.

### *Flcn* loss activates autophagy in a mouse model of liver fibrosis

**Figure 4:**
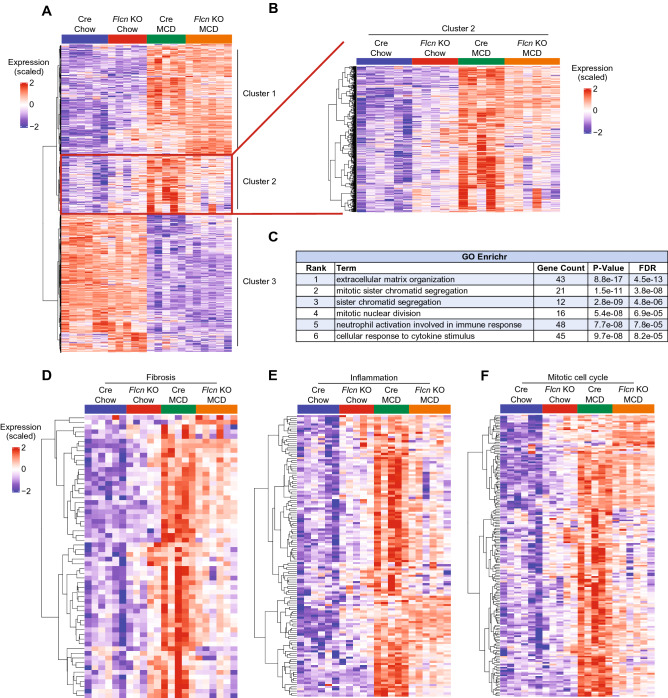
RNA-seq reveals major pathways affected by *Flcn* loss. (**A,B**) Unsupervised hierarchical clustering following RNA-seq analysis in livers of mice fed either chow or methionine/choline deficient diet (MCD) over a period of 6 weeks. (**C**) Gene enrichment analysis using GO Enrichr. (**D**) Unsupervised hierarchical clustering following RNA-seq analysis of fibrosis-related genes (GO:0030198). (**E**) Unsupervised hierarchical clustering following RNA-seq analysis of inflammation-related genes (GO:0006954). (**F**) Unsupervised hierarchical clustering following RNA-seq analysis of mitotic cell cycle-related genes (GO:0000278).

This protective impact on inflammation and fibrosis following loss of *Flcn* prompted us to investigate the molecular signature of liver-*Flcn*^−/−^ mice responsible for the protective phenotype using high-throughput whole-transcriptome sequencing (mRNA-seq). Unsupervised hierarchical clustering highlighted three distinct patterns of gene expression changes (Fig. 4A, Supplemental Table 1), clusters 1 and 3 not being affected by the *Flcn* status. Interestingly, cluster 2 highlighted genes whose expression was induced in Cre mice receiving the MCD diet but that were not increased in liver-*Flcn*^−/−^ mice (Fig. 4A,B). Gene enrichment analysis using GO Enrichr revealed the major pathways differentially affected in this cluster by the diet and the genotypes (Fig. 4C). The most significantly affected biological pathway was extracellular matrix organization, which represents genes involved in fibrosis development typically observed in NASH, such as *Acta2*, *Mmp2*, *Serpine1*, and *Timp1* (as validated by qRT-PCR and consistent with histology experiments in Fig. 3). Other important pathways affected include mitotic sister chromatid segregation, sister chromatic segregation, and mitotic nuclear division. These genes are generally induced in regenerating livers following injury^29^. Moreover, pathways involved in inflammation such as neutrophil activation and cellular response to cytokine stimulus were differentially expressed. Overall, genes involved in fibrosis, inflammation and mitotic cell cycle were induced in mice fed the MCD diet but to a lesser extent in *Flcn* KO mice (Fig. 4D–F). Concurrently, no significant changes in expression between Cre and *Flcn* KO mice were detected for pathways involved in lipolysis (GO:0016042) and lipogenesis (GO:0008610) (Supplemental Fig. 3A–C).

Emerging data supports a role of autophagy in the liver to regulate intracellular lipid stores and energy homeostasis. Autophagy is an important process in which the cells degrade its own content by sequestering cargoes inside the lysosomes. Degradation of these cargoes inside the lysosome is a multistep process that can be regulated by master transcription factors such as Transcription Factor EB (TFEB) and Transcription Factor Binding To IGHM Enhancer 3 (TFE3)^15,30^. The best described regulator of TFEB and TFE3 is the mTOR Complex 1 (mTORC1). Under non-stress conditions, mTORC1 phosphorylates TFEB and TFE3, resulting in cytoplasmic sequestration and inhibition of their activity^15^. Conversely, nutrient starvation induced inhibition of mTORC1 removes the repressive phosphorylation on TFEB and TFE3, resulting in their nuclear translocation and activation of a panel of genes involved in autophagy^15^. We and others have shown that FLCN is a negative regulator of TFEB and TFE3 and positive regulator of mTORC1^23,31–33^. Moreover, FLCN exhibits GTPase-activating protein (GAP) activity upon nutrient replenishment targeting the Ras-related GTPase C and Ras-related GTPase D (RagC/D) resulting in mTORC1 activation, inhibition of TFEB and TFE3 nuclear translocation, and impaired autophagy and lysosomal function^24,25^.

**Figure 5:**
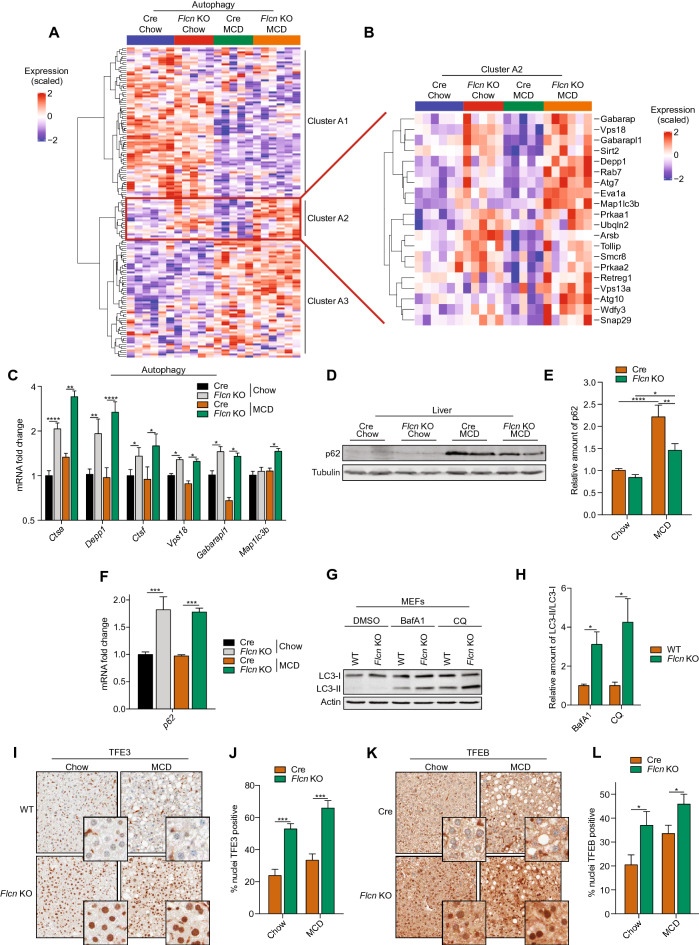
*Flcn* loss activates autophagy in a mouse model of liver fibrosis. (**A-B**) Unsupervised hierarchical clustering following RNA-seq analysis in livers of mice fed either on chow or methionine/choline deficient diet (MCD) over a period of 6 weeks. (**C**) Relative quantitative real‐time PCR analysis of autophagy-related genes mRNA transcript levels in livers of mice fed as described in (**A**) (mean ± SEM of the RNA fold change of indicated mRNAs; two-way ANOVA, *P < 0.05, **P < 0.01, ****P < 0.0001; n = 8 mice per condition). (**D**) Immunoblot of liver protein lysates extracted from mice fed as described in (**A**). Data are representative of 6 mice per condition. Full-length blots are presented in Supplementary Fig. 5. (**E**) Quantification of the relative amount of p62 immunoblot. Data normalized to tubulin levels (mean ± SEM of the relative fold change; two-way ANOVA, *P < 0.05, **P < 0.01, ****P < 0.0001; n = 6 mice per condition). (**F**) Relative RNAseq analysis of p62 mRNA transcript levels in livers of mice fed as described in (**A**) (mean ± SEM of p62 RNA fold change; one-way ANOVA, ***P < 0.001; n = 5 mice per condition). (**G**) Immunoblot of protein lysates of WT and *Flcn* KO MEFs incubated in presence of DMSO, BafA1 (100 nM), or Chloroquine (CQ; 100 μM) for 6 h. Data are representative of three independent experiments. Full-length blots are presented in Supplementary Fig. 5. (**H**) Quantification of the relative amount of LC3-II/LC3-I immunoblot of MEFs treated as described in (G) (mean ± SEM of three independent experiment, one-way ANOVA, *P < 0.05). (**I**) TFE3 Immunohistochemistry (IHC) staining in liver sections of mice fed as described in (**A**) Data are representative of 6 mice per condition. (**J**) Quantification of TFE3 IHC staining in liver sections of mice fed as described in (**A**) (mean ± SEM, two-way ANOVA, ***P < 0.001; n = 4 mice per condition). (**K**) TFEB Immunohistochemistry (IHC) staining in liver sections of mice fed as described in (**A**) Data are representative of 4 mice per condition. (**L**) Quantification of TFEB IHC staining in liver sections of mice fed as described in (**A**) (mean ± SEM, two-way ANOVA, ***P < 0.001; n = 4 mice per condition).

Unsupervised hierarchical clustering of autophagy-related genes from our RNA-seq data revealed an important effect of the diet (Autophagy clusters A1 and A3, Fig. 5A), but also highlighted a subset of genes induced in liver-*Flcn*^−/−^ mice (Autophagy cluster A2, Fig. 5A-B). Relative mRNA transcript levels of autophagy-related genes mRNA, such as *Ctsa*, *Depp1*, and *Ctsf*, were significantly increased in liver-*Flcn*^−/−^ mice compared to Cre mice, while the diet had no effect (Fig. 5C). These genes are generally involved in lysosome/autophagosome maturation and activation, which can reduce hepatocellular injury and inflammation by clearing damaged or misfolded proteins and suppressing transcription or maturation of pro-inflammatory cytokines^34^. Immunoblot analysis additionally revealed higher protein levels of p62 in Cre mice challenged with the MCD diet, suggesting impaired autophagy, while *Flcn* loss reduced p62 protein levels (Fig. 5D, quantified in E). p62 mRNA transcript levels were induced in liver-*Flcn* KO but were unaffected by the diet (Fig. 5F). Therefore, the change in p62 protein levels is unlikely to be due to regulation at the mRNA level of p62. To further assess autophagy induction in a *Flcn* KO model, we measured autophagy flux in mouse embryonic fibroblasts (MEFs) in the absence and presence of BafA1 and Chloroquine, two compounds known to pharmacologically block autophagosome-lysosome fusion. *Flcn* loss in this cell line increased autophagy flux upon autophagy blockade, as measured by higher LC3-II accumulation in *Flcn* KO after treatment with BafA1 and Chloroquine (Fig. 5G, quantified in H). We also measured LC3 levels in liver protein lysates from mice fed either the chow or the MCD diet. We show that *Flcn* KO mice had higher LC3-I and LC3-II levels in both diets, suggesting functional and active autophagy (Supplemental Fig. 4A, quantified in B and C). Furthermore, the major transcription factors regulating autophagy and lysosome biogenesis, TFE3 and TFEB, were localized in the nuclei of hepatocytes, which correlated with increased autophagy in *Flcn* KO mice (Fig. 5I–L). Taken together, our data suggest that targeting *Flcn* improves resistance to fibrosis and inflammation, possibly by inducing autophagy when challenged with a liver fibrosis-inducing diet.

## Discussion

In this study, we have engineered a mouse model to specifically ablate *Flcn* in the liver to elucidate the potential key role of this factor in the control of hepatic metabolism. Interestingly, liver specific loss of *Flcn* markedly enhanced whole-body energy metabolism at the baseline level as shown by the increased energy expenditure as well as improved glucose and insulin tolerance in these mice under HFD. Remarkably, deletion of *Flcn* in mouse liver tissue reduced fibrosis and inflammation when fed the fibrosis inducing MCD diet. Overall, our results establish a critical role of *Flcn* in liver-fibrosis progression.

Our work has highlighted for the first time a protective role for *Flcn* loss in the liver. Similarly, *Flcn* deletion in other tissues such as adipocytes and muscles led to improved capabilities and whole-body metabolism homeostasis^21,22^. The protective phenotype observed in the liver is comparable to previous attempts to activate autophagy and inhibit mTORC1 with rapalogs and rapamycin^13,14^, but was not accompanied by the deleterious long-term effects of these compounds on liver damage and tumorigenesis^17,18^. Indeed, we did not observe any harmful effect of *Flcn* deletion in livers. The difference is possibly the result of the more specific regulation of mTORC1 by FLCN on the lysosomal surface that is not affecting the mRNA translation machinery^23,26^. On the other hand, rapalogs regulate all mTOR complexes in the cell, reinforcing the potential therapeutic benefit of targeting more precisely FLCN.

Under the fibrosis-inducing diet, which mimics fibrosis as observed with NASH, RNA-seq data revealed that major pathways affected by *Flcn* loss specifically in the liver include inflammation, fibrosis, and mitotic cell cycle gene expression. The reduction in cell cycle gene expression is possibly the consequence of reduced liver damage and therefore of the reduced necessity to regenerate liver cells. We have previously shown that loss of *Flcn* in *C. elegans* and mammalian cells increased innate immune response in a process dependent on TFEB and TFE3^32^. In this study, we observed a decrease in inflammation gene expression in liver-*Flcn* KO mice fed the MCD diet. It is possible that FLCN modulates the immune microenvironment and polarizes the macrophages toward an anti-inflammatory state. Indeed, it was reported that *FLCN* loss in hematopoietic stem cells induced CD11c + CD206 + activated phagocytic macrophages, corresponding to the anti-inflammatory M2 macrophage subtype^35^. However, another study found that bone marrow-derived macrophages from *Flcn* myeloid KO mice rather promotes spontaneous M1-type polarization and enhanced baseline activation status^33^. Also, RAW 264.7 macrophages targeted with shRNA for *Flcn* had increased phagocytic activity toward dying/dead cells^36^. The previous effects of *Flcn* loss on macrophage activation were all dependent on TFEB/TFE3 activation status, highlighting the important role of autophagy in FLCN’s mechanism of regulation. Therefore, the importance and significance of the inflammation observed in *Flcn* KO livers remains to be investigated.

FLCN seemed to be involved in regulating autophagy in a severe liver disease setting and it is possibly the primary mechanism of action by which loss of *Flcn* reduces liver fibrosis. Several compounds known to activate autophagy pathways such as acetylshikonin and resveratrol also reduced MCD-induced fibrosis and inflammation^37,38^. Acetylshikonin contributed to the removal of cellular lipid droplets through lipophagy in a process dependent on the AMPK/mTOR pathways^37^. Indeed, acute inhibition of mTOR and activation of autophagy with rapamycin in mice reduced liver injury and inflammation similarly to our study^39^. Moreover, thioredoxin interacting protein (TXNIP/VDUP1) mediates the activation of AMPK, inhibition of mTOR, and nuclear translocation of TFEB which ultimately resulted MCD diet-induced steatosis, inflammation, and fibrosis^40^.

In conclusion, we revealed that loss of *Flcn* in the liver is involved in autophagy, resulting in reduction of inflammation, fibrosis, and enhanced whole-body energy metabolism. These results show an unexpected role for *Flcn* in NAFLD and NASH progression. In light of the recently published cryo-EM structures of FLCN^41,42^, it is envisioned that pharmacological agents targeting the pocket of the FLCN GAP enzyme could be developed to achieve the loss of function phenotype in the liver shown in this study. Such molecules would represent new possibilities for treatment of obesity and NASH through the role of FLCN in hepatocyte homeostasis.

## Material and methods

### Antibodies

The FLCN rabbit polyclonal antibody was generated by the McGill Animal Resource Centre using recombinant GST-FLCN. TFEB (Bethyl Laboratories, cat# A303-673A), TFE3 (Cell Signaling Technology, cat# 14779S), α-SMA (Abcam, cat# 5694), F4/80 (Cell Signaling Technology, cat# 70,076), 4-HNE (Millipore, cat# 393,207), p62 (Abcam, cat# ab56416), LC3B (Abcam, cat# ab48934), and Tubulin (Sigma-Aldrich, Cat# T9026) antibodies are commercially available.

### Animals

All procedures for generating the *Flcn* knockout mouse model were performed at McGill University. Maintenance and experimental manipulation of mice were performed according to the guidelines and regulations of the McGill University Research and Ethic Animal Committee and the Canadian Council on Animal Care. The study was carried out in compliance with the ARRIVE guidelines and all experimental protocols were approved by the McGill University Research and Ethic Animal Committee. All studies were carried out using C57BL/6 mice housed on a 12 h light:12 h dark cycle at 22 °C. *Flcn*^f/f^ (BHD^f/f^) mice were generated as previously described^22^. To generate liver-specific *Flcn* knockout mice, *Flcn*^f/f^ mice were crossed with Albumin-Cre transgenic mice (kind gift from Dr. André Marette, Laval University, Quebec, Canada). Liver-*Flcn*^−/−^ knockout mice were generated by crossing *Flcn*^f/f^ mice homozygous for the floxed allele with *Flcn*^f/f^; Albumin-Cre^+/−^ mice. Mice were fed on a chow diet containing 10 kcal percent fat (Research Diet, cat# D12450B) or a high fat diet containing 60 kcal percent fat (Research Diet, cat# D12492), or a Methionine/Choline Deficient (MCD) diet (Envigo, cat# TD.90262) beginning at 6 weeks of age and fed ad libidum. Controls used were *Flcn*^WT/WT^; Albumin-cre^+/−^ mice raised in identical conditions. Body weights were measured weekly.

### Cell lines culture and treatments

Primary MEFs were isolated from C57BL/6 E12.5 *Flcn* floxed mice (generously provided by Dr. L.S. Schmidt, NCI, Bethesda, MD, USA) and cultured as described previously^19^. FLCN KO MEFs were generated after immortalization of primary Flox/Flox MEFs with SV40 large T and retroviral infection with a Cre recombinase, followed by puromycin selection. Cell lines were maintained in Dulbecco's Modified Eagle's Medium (DMEM) supplemented with 10% fetal bovine serum (FBS), 100 U/ml penicillin, 100 µg/ml streptomycin, and 50 µg/mL gentamycin (Wisent) in 5% CO_2_ at 37 °C. For drug treatment experiments, cells were incubated for 6 h in medium containing one of the following reagents: DMSO (0.4%, Bioshop Canada), Chloroquine (100 μM, Sigma-Aldrich), and BafA1 (100 nM, Sigma-Aldrich).

### Protein extraction and immunoblotting

Liver tissues were quickly snap frozen using a BioSpec BioSqueezer (Fisher Scientific, cat# NC1033496) cooled in liquid nitrogen following sacrifice. Approximately 100 mg of tissue were solubilized in RIPA buffer using a micropestle and sonicated 10 s. The mixture was then centrifugated at 16,000 × *g* for 15 min at 4 °C and the supernatant collected. Proteins were separated on SDS-PAGE gels and revealed by western blot as previously described^22^ using the antibodies listed above and IRDye 800CW Goat anti-Rabbit IgG Secondary Antibody / IRDye 680RD Goat anti-Mouse IgG Secondary Antibody (LI-COR Biosciences). Membranes were scanned using the Odyssey imaging system (LI-COR Biosciences) and quantification performed using ImageStudio software (version 5.2.5, https://www.licor.com/bio/image-studio-lite/).

### Histology

All histological procedures were performed using standard procedures at the histology core facility of the Goodman Cancer Research Centre (McGill University). Liver tissues were fixed in 4% formaldehyde for 2 days at room temperature immediately after sacrifice, embedded in paraffin, and cut to 4-μm sections on slides using a Leica Microtome (Leica Biosystems). The slides were stained with hematoxylin and eosin (Leica Biosystems, cat# 3802098), or Sirius Red (Abcam, cat# ab150681) using a Leica ST5020 Multistainer (Leica Biosystems) according to the standard protocol. Stained images were acquired using an Aperio ScanScope XT digital scanner (Leica Biosystems) and analyzed with ImageScope software (version 12.3.3.5048, https://www.leicabiosystems.com/fr/imagerie-pathologique/integrer/aperio-imagescope/).

### Metabolic cages

Mice were housed individually in metabolic cages for a total 3 days on a 12 h light: 12 h dark cycle with free access to water and food using a comprehensive laboratory animal monitoring system (CLAMS; Columbus Instruments). Food intake, energy expenditure, physical activity, maximal carbon dioxide (CO_2_) production (VCO_2_) and oxygen (O_2_) consumption (VO_2_) rates were measured simultaneously using recorded values from a 72 h period.

### GTTs and ITTs

For the GTTs, mice on either chow or a HFD were subjected to a 16 h fast with free access to water and then injected intraperitoneally with 2 g of 20% d-glucose per kilogram of body weight. The ITTs were performed similarly, with an initial 16 h of fasting and subsequent intraperitoneal injection of 0.75 U of insulin per kilogram of body weight. Blood glucose levels were measured at 0, 15, 30, 60, and 120 min with a OneTouch ultramini glucose meter (OneTouch).

### Serum content quantification

Blood was extracted from cardiac puncture just before sacrifice and incubated with Aprotinin from bovine lung (0.03 TIU, Sigma-Aldrich, cat# A6279) for 2 h at 4 °C. The coagulated blood was spun 10 min at 800 × *g* at 4 °C and the supernatant (serum) stored at -80 °C. Serum total cholesterol, serum free cholesterol, and serum cholesterol esters were quantified using Cholesterol/ Cholesteryl Ester Assay Kit (Abcam, cat# ab65359) according to manufacturer’s instructions. Serum Triglyceride and serum free glycerol were quantified using Serum Triglyceride Determination Kit (Sigma-Aldrich, cat# TR0100) according to manufacturer’s instructions. Serum alanine aminotransferase was quantified using ALT determination kit (Pointe Scientific, cat# A7526) according to manufacturer’s instructions.

### Liver triglyceride quantification

Approximately 10 mg of frozen liver tissues were washed with PBS, resuspended in a 2:1 chloroform:methanol solution, and solubilized with a micropestle. The mixture was spun for 10 min at 15,000 × *g* at 4 °C and the supernatant incubated at 50 °C until the chloroform was completely evaporated. The dried pellet was dissolved in a solution containing 60% Butanol, 25% Triton X-100, and 15% Methanol and quantified using Serum Triglyceride Determination Kit (Sigma-Aldrich, cat# TR0100) according to manufacturer’s instructions.

### Quantitative real-time PCR

Total RNA was isolated and purified from approximately 25 mg of frozen liver tissue or cells using Total RNA Mini Kit (Geneaid, cat# RT100) according to the manufacturer's instructions. For quantitative real-time PCR analysis, 0.5 μg of total RNA was reverse-transcribed using the iScript Reverse Transcription Supermix for RT-qPCR (BioRad, cat# 1708841). SYBR Green reactions using the SYBR Green qPCR Supermix (BioRad, cat# 1,725,125) and specific primers (Table 1) were performed using a CFX Connect Real-Time PCR Detection System (BioRad). Relative expression of mRNAs was determined using the software CFX Maestro (BioRad, version 4.1.2434.0124, https://www.bio-rad.com/en-ca/product/cfx-maestro-software-for-cfx-real-time-pcr-instruments?ID=OKZP7E15) after normalization against housekeeping genes B2M, TBP, RPLP0, and PTP4a1.

**Table 1:** List of mouse primers used in RT-qPCR experiments.

Gene	Forward primers	Reverse primers
ACTA2	GACTCACAACGTGCCTATC	GCAGTAGTCACGAAGGAATAG
AKR1A1	CTGGAGTATTTGGACCTCTATTT	AGCCTTCCAGGTCTCTTTA
ALDOA	GTGGTGTTGTGGGCATTA	TCGGCTCCATCCTTCTTAT
APOA4	GACCACGATCAAGGAGAATG	CCCTTGAGCTCTTCCATATTC
APOE	GGAACAGACCCAGCAAATAC	CTGTATCTTCTCCATCAGGTTTG
ASAH1	ACCGGCCAAGAAGTGTCTAA	GGTCTGGGCAATCTCGAAGG
ATP5D	GACGCAGGTGTTCTTTGA	CTTCTGTGTGAACCACTACC
B2M	CTGACCGGCCTGTATGCTAT	CCGTTCTTCAGCATTTGGAT
BAX	GAGATGAACTGGACAGCAATA	AGTAGAAGAGGGCAACCA
BCL2	GGTCCCACACTGAATAGAATAG	AGAGACACCCATCACCTT
C5AR1	GCTAGGCAGATACACCTAATG	AGAAGAGGAAGAGAGGAAGAA
CASP1	GTGGGACCACATACTCTAATG	CACGGCATGCCTGAATAA
CCL2	GAATGGGTCCAGACATACATTA	TACGGGTCAACTTCACATTC
CCL5	CCAGAGAAGAAGTGGGTTCAAG	AGCAATGACAGGGAAGCTATAC
CD36	GGATGGTTTCCTAGCCTTTC	TGGCCCGGTTCTACTAAT
CTSA	GAGCAGAACGACAACTCCCT	TGCCCACAATTCGAGACACT
CTSF	GTTGCCATTAACGCCTTCGG	TTAGAGCGGTTGCCATAGCC
CXCL1	CAACCACTGTGCTAGTAGAAG	CATGTCCTCACCCTAATACATAC
FAS	CTCCAGTCGTGAAACCATAC	CTTGCCCTCCTTGATGTTAT
FGF21	CCAAGACCAAGCAGGATTC	AGCCCTAGATTCAGGAAGAG
G6PC	CAGCTCCGTGCCTATAATAAA	GCAAGAGTAGAAGTGACCATAA
IL-1β	GGGCAACCACTTACCTATTT	GGGCTATGACCAATTCATCC
LCN2	GTCTGCCACTCCATCTTTC	GGAGTGCTGGCCAAATAA
LGALS3	CTAACCACGCCATGATCTAAG	ACAAGAAGGATAAGGAGAGAGA
MCOLN1	CCACCACGGACATAGGCATAC	GCTGGGTTACTCTGATGGGTC
MMP2	GAGGACTATGACCGGGATAA	TGGTGCAGCTCTCATACT
NEU1	CAGAGATGTTTGCCCCTGGA	GACAGAAGACCCCATCTCGC
PEPCK	GATGTCGGAAGAGGACTTTG	CGAGTCTGTCAGTTCAATACC
SDHA	TTACCTGCGTTTCCCCTCAT	AAGTCTGGCGCAACTCAATC
SERPINE1	AGAGAGAGAGATTTGAGAGAGG	GGAGAAAGTTCTGTCCTGTTAG
SOCS3	GGGTGGCAAAGAAAAGGAG	GTTGAGCGTCAAGACCCAGT
TBP	ACCTTATGCTCAGGGCTTGG	GCCATAAGGCATCATTGGAC
TGFβ1	TCAGCTCCACAGAGAAGAA	GTGTCCAGGCTCCAAATATAG
TIMP1	AATCAACGAGACCACCTTATAC	CATATCCACAGAGGCTTTCC
TNFα	GGGTGTTCATCCATTCTCTAC	TGGACCCTGAGCCATAAT
UQCRC2	TTCGTTAAAGCAGGCAGTAG	CTTCAATCCCACGGGTTATC
VLCAD	CATCCTCAACAACGGAAGAT	CCCAAACTGGGTACGATTAG

### Immunohistochemistry

Liver tissues embedded in paraffin were cut to 4-μm sections on slides and stained for α-SMA, 4-HNE, F4/80, TFE3 and TFEB using routine immunohistochemical protocols provided by the GCRC Histology Core using Ventana BenchMark ULTRA system (Roche). Images were acquired using Aperio Scanscope XT (Leica Biosystems) and staining quantification was performed using image analysis algorithms in Aperio ImageScope software (version 12.3.3.5048, https://www.leicabiosystems.com/fr/imagerie-pathologique/integrer/aperio-imagescope/).

### Mouse protein cytokine array

Approximately 100 mg of liver tissue were solubilized in RIPA buffer using a micropestle and sonicated 10 s. The mixture was then centrifugated at 16,000 × g for 15 min at 4 °C and the supernatant collected. 31 mouse cytokine/chemokine biomarkers were simultaneously quantified by using a Discovery Assay called the Mouse Cytokine Array / Chemokine Array 31-Plex (Eve Technologies Corp, cat# MD31).

### RNA-seq analysis

Total RNA was isolated and purified from approximately 25 mg of frozen liver tissue using Total RNA Mini Kit (Geneaid, cat# RT100) according to the manufacturer's instructions. Library preparation and sequencing was made at the Institute for Research in Immunology and Cancer’s Genomics Platform (IRIC). 500 ng of total RNA was used for library preparation. RNA quality control was assessed with the Bioanalyzer RNA 6000 Nano assay on the 2100 Bioanalyzer system (Agilent Technologies). Library preparation was done with the KAPA mRNAseq Hyperprep kit (KAPA, cat# KK8581). Ligation was made with Illumina dual-index UMI (IDT) and 10 PCR cycles was required to amplify cDNA libraries. Libraries were quantified by QuBit and BioAnalyzer DNA1000. All libraries were diluted to 10 nM and normalized by qPCR using the KAPA library quantification kit (KAPA, cat# KK4973). Libraries were pooled to equimolar concentration. Sequencing was performed with the Illumina Nextseq500 using the Nextseq High Output 75 (1 × 75 bp) cycles kit using 2.6 pM of the pooled libraries. Around 25 M single-end PF reads were generated per sample.

Following data acquisition, adaptor sequences and low quality score bases (Phred score < 30) were first trimmed using Trimmomatic^43^. The resulting reads were aligned to the GRCm38 mouse reference genome assembly, using STAR^44^, and read counts were obtained using HTSeq^45^. For all downstream analyses, we excluded lowly-expressed genes with an average read count lower than 10 across all samples, resulting in 14,057 expressed genes in total. The R package limma^46^ was used to identify differences in gene expression levels between the different conditions. Nominal p-values were corrected for multiple testing using the Benjamini–Hochberg method. To assess the effect liver-*Flcn* KO in the response to MCD-diet, we first obtained differentially expressed genes (FDR < 0.05 and |log2FC|> 1) in Cre.MCD_diet vs Cre.Chow and *Flcn*-KO.MCD_diet vs *Flcn*-KO.Chow, and then filtered for those that show |difference in log2FC|> 1 (differentially responsive (DR) genes). Unsupervised hierarchical clustering of the 1,110 DR genes shows three distinct patterns of changes in expression. Pathway enrichment analyses were performed using Enrichr^47^.

### Statistical analyses

Data are expressed as mean ± SEM. All experiments and measurements were performed on at least 3 mice as indicated. Statistical analyses for all data were performed using student's t-test, one-way ANOVA or two-way ANOVA as indicated using GraphPad Prism 7 software (version 7.0a, https://www.graphpad.com/scientific-software/prism/). Statistical significance is indicated in figures (*P < 0.05, **P < 0.01, ***P < 0.001, ****P < 0.0001).

## Supplementary Information

Supplementary Information 1.

Supplementary Information 2.

## Data Availability

RNA-sequencing data has been deposited in the Gene Expression Omnibus under the accession GSE156918.

## Abbreviations

FLCN: Folliculin
HFD: High fat diet
H&E: Hematoxylin and eosin
MCD: Methionine/Choline Deficient diet
mTORC1: Mammalian Target of Rapamycin Complex 1
NAFLD: Non-alcoholic fatty liver disease
NASH: Non-alcoholic steatohepatitis
TFEB: Transcription Factor EB
TFE3: Transcription Factor Binding To IGHM Enhancer 3

## References

[1] European Association for the Study of the Liver (EASL), European Association for the Study of Diabetes (EASD), European Association for the Study of Obesity (EASO). EASL-EASD-EASO Clinical Practice Guidelines for the Management of Non-Alcoholic Fatty Liver Disease. Obes. Facts. 2016;9:65–90. doi:10.1159/000443344. 10.1159/000443344 PMC5644799 27055256

[2] Arab JP, Arrese M, Trauner M (2018). Recent insights into the pathogenesis of nonalcoholic fatty liver disease. Annu. Rev. Pathol..

[3] Satapathy SK, Sanyal AJ (2015). Epidemiology and natural history of nonalcoholic fatty liver disease. Semin. Liver Dis..

[4] Angulo P, Kleiner DE, Dam-Larsen S, Adams LA, Bjornsson ES, Charatcharoenwitthaya P (2015). Liver fibrosis, but no other histologic features, is associated with long-term outcomes of patients with nonalcoholic fatty liver disease. Gastroenterology.

[5] Younossi ZM (2019). Non-alcoholic fatty liver disease—A global public health perspective. J. Hepatol..

[6] Wong VW-S, Adams LA, de Lédinghen V, Wong GL-H, Sookoian S (2018). Noninvasive biomarkers in NAFLD and NASH - current progress and future promise. Nat. Rev. Gastroenterol. Hepatol..

[7] Ibrahim SH, Hirsova P, Gores GJ (2018). Non-alcoholic steatohepatitis pathogenesis: sublethal hepatocyte injury as a driver of liver inflammation. Gut.

[8] Machado MV, Diehl AM (2016). Pathogenesis of nonalcoholic steatohepatitis. Gastroenterology.

[9] Ashraf NU, Sheikh TA (2015). Endoplasmic reticulum stress and Oxidative stress in the pathogenesis of Non-alcoholic fatty liver disease. Free Radic. Res..

[10] Angulo P, Machado MV, Diehl AM (2015). Fibrosis in nonalcoholic Fatty liver disease: mechanisms and clinical implications. Semin. Liver Dis..

[11] Machado MV, Diehl AM (2014). Liver renewal: detecting misrepair and optimizing regeneration. Mayo Clin. Proc..

[12] Hardie DG, Schaffer BE, Brunet A (2016). AMPK: An Energy-Sensing Pathway with Multiple Inputs and Outputs. Trends Cell Biol..

[13] Chen H, Shen F, Sherban A, Nocon A, Li Y, Wang H (2018). DEP domain-containing mTOR-interacting protein suppresses lipogenesis and ameliorates hepatic steatosis and acute-on-chronic liver injury in alcoholic liver disease. Hepatology.

[14] Lin C-W, Zhang H, Li M, Xiong X, Chen X, Chen X (2013). Pharmacological promotion of autophagy alleviates steatosis and injury in alcoholic and non-alcoholic fatty liver conditions in mice. J. Hepatol..

[15] Paquette M, El-Houjeiri L, Pause A (2018). mTOR Pathways in Cancer and Autophagy. Cancers (Basel).

[16] He A, Dean JM, Lu D, Chen Y, Lodhi IJ (2020). Hepatic peroxisomal β-oxidation suppresses lipophagy via RPTOR acetylation and MTOR activation. Autophagy.

[17] Umemura A, Park EJ, Taniguchi K, Lee JH, Shalapour S, Valasek MA (2014). Liver damage, inflammation, and enhanced tumorigenesis after persistent mTORC1 inhibition. Cell Metab.

[18] Yamanaka K, Petrulionis M, Lin S, Gao C, Galli U, Richter S (2013). Therapeutic potential and adverse events of everolimus for treatment of hepatocellular carcinoma - systematic review and meta-analysis. Cancer Med.

[19] Possik E, Jalali Z, Nouët Y, Yan M, Gingras M-C, Schmeisser K (2014). Folliculin regulates ampk-dependent autophagy and metabolic stress survival. PLoS Genet..

[20] Possik E, Ajisebutu A, Manteghi S, Gingras M-C, Vijayaraghavan T, Flamand M (2015). FLCN and AMPK confer resistance to hyperosmotic stress via remodeling of glycogen stores. PLoS Genet..

[21] Hasumi H, Baba M, Hasumi Y, Huang Y, Oh H, Hughes RM (2012). Regulation of mitochondrial oxidative metabolism by tumor suppressor FLCN. J Natl. Cancer Inst..

[22] Yan M, Audet-Walsh É, Manteghi S, Dufour CR, Walker B, Baba M (2016). Chronic AMPK activation via loss of FLCN induces functional beige adipose tissue through PGC-1α/ERRα. Genes Dev..

[23] Wada S, Neinast M, Jang C, Ibrahim YH, Lee G, Babu A (2016). The tumor suppressor FLCN mediates an alternate mTOR pathway to regulate browning of adipose tissue. Genes Dev..

[24] Meng J, Ferguson SM (2018). GATOR1-dependent recruitment of FLCN-FNIP to lysosomes coordinates Rag GTPase heterodimer nucleotide status in response to amino acids. J. Cell Biol..

[25] Tsun Z-Y, Bar-Peled L, Chantranupong L, Zoncu R, Wang T, Kim C (2013). The folliculin tumor suppressor is a GAP for the RagC/D GTPases that signal amino acid levels to mTORC1. Mol. Cell.

[26] Napolitano G, Di Malta C, Esposito A, de Araujo MEG, Pece S, Bertalot G (2020). A substrate-specific mTORC1 pathway underlies Birt-Hogg-Dubé syndrome. Nature.

[27] Van Herck MA, Vonghia L, Francque SM (2017). Animal models of nonalcoholic fatty liver disease—A Starter’s guide. Nutrients.

[28] Itagaki H, Shimizu K, Morikawa S, Ogawa K, Ezaki T (2013). Morphological and functional characterization of non-alcoholic fatty liver disease induced by a methionine-choline-deficient diet in C57BL/6 mice. Int. J. Clin. Exp. Pathol..

[29] Caldez MJ, Bjorklund M, Kaldis P (2020). Cell cycle regulation in NAFLD: when imbalanced metabolism limits cell division. Hepatol. Int..

[30] Sardiello M, Palmieri M, di Ronza A, Medina DL, Valenza M, Gennarino VA (2009). A gene network regulating lysosomal biogenesis and function. Science.

[31] Hong S-B, Oh H, Valera VA, Baba M, Schmidt LS, Linehan WM (2010). Inactivation of the FLCN tumor suppressor gene induces TFE3 transcriptional activity by increasing its nuclear localization. PLoS ONE.

[32] El-Houjeiri L, Possik E, Vijayaraghavan T, Paquette M, Martina JA, Kazan JM (2019). The Transcription Factors TFEB and TFE3 Link the FLCN-AMPK Signaling Axis to Innate Immune Response and Pathogen Resistance. Cell Rep..

[33] Li J, Wada S, Weaver LK, Biswas C, Behrens EM, Arany Z (2019). Myeloid Folliculin balances mTOR activation to maintain innate immunity homeostasis. JCI Insight.

[34] Lee H-M, Shin D-M, Yuk J-M, Shi G, Choi D-K, Lee S-H (2011). Autophagy negatively regulates keratinocyte inflammatory responses via scaffolding protein p62/SQSTM1. J. Immunol..

[35] Endo M, Baba M, Endoh T, Umemoto T, Hashimoto M, Chong Y (2017). The FLCN-TFE3 axis regulates macrophage activation through cellular metabolism. Exp. Hematol..

[36] Endoh M, Baba M, Endoh T, Hirayama A, Nakamura-Ishizu A, Umemoto T (2020). A FLCN-TFE3 feedback loop prevents excessive glycogenesis and phagocyte activation by regulating lysosome activity. Cell Rep..

[37] Zeng J, Zhu B, Su M (2018). Autophagy is involved in acetylshikonin ameliorating non-alcoholic steatohepatitis through AMPK/mTOR pathway. Biochem. Biophys. Res. Commun..

[38] Ji G, Wang Y, Deng Y, Li X, Jiang Z (2015). Resveratrol ameliorates hepatic steatosis and inflammation in methionine/choline-deficient diet-induced steatohepatitis through regulating autophagy. Lipids Health Dis..

[39] Chen R, Wang Q, Song S, Liu F, He B, Gao X (2016). Protective role of autophagy in methionine-choline deficient diet-induced advanced nonalcoholic steatohepatitis in mice. Eur. J. Pharmacol..

[40] Park H-S, Song J-W, Park J-H, Lim B-K, Moon O-S, Son H-Y (2020). TXNIP/VDUP1 attenuates steatohepatitis via autophagy and fatty acid oxidation. Autophagy.

[41] Shen K, Rogala KB, Chou H-T, Huang RK, Yu Z, Sabatini DM (2019). Cryo-EM Structure of the Human FLCN-FNIP2-Rag-Ragulator Complex. Cell.

[42] Lawrence RE, Fromm SA, Fu Y, Yokom AL, Kim DJ, Thelen AM (2019). Structural mechanism of a Rag GTPase activation checkpoint by the lysosomal folliculin complex. Science.

[43] Bolger AM, Lohse M, Usadel B (2014). Trimmomatic: a flexible trimmer for Illumina sequence data. Bioinformatics.

[44] Dobin A, Davis CA, Schlesinger F, Drenkow J, Zaleski C, Jha S (2013). STAR: ultrafast universal RNA-seq aligner. Bioinformatics.

[45] Anders S, Pyl PT, Huber W (2015). HTSeq — A Python framework to work with high-throughput sequencing data. Bioinformatics.

[46] Ritchie ME, Phipson B, Wu D, Hu Y, Law CW, Shi W (2015). limma powers differential expression analyses for RNA-sequencing and microarray studies. Nucleic Acids Res.

[47] Kuleshov MV, Jones MR, Rouillard AD, Fernandez NF, Duan Q, Wang Z (2016). Enrichr: a comprehensive gene set enrichment analysis web server 2016 update. Nucleic Acids Res.

