# Supplementary material for: Loss of hepatic Flcn protects against fibrosis and inflammation by activating autophagy pathways
Source: Sci Rep. 2021 Oct 28;11:21268. doi: 10.1038/s41598-021-99958-7 (PMC8553785; doi:10.1038/s41598-021-99958-7)
Supplement: Supplementary file 2 — Supplementary Information 2. [file 41598_2021_99958_MOESM2_ESM.pdf]

Supplementary material for manuscript:

Loss of *Flcn* protects against fibrosis and inflammation by activating autophagy pathways

Authors/Affiliation

Mathieu Paquette[1,2], Ming Yan[1,2], Josué M. J. Ramírez-Reyes[1,2], Leeanna El-Houjeiri[1,2], Marco Biondini[1,2], Catherine R. Dufour[1,2], Hyeonju Jeong[1,2], Alain Pacis[3], Vincent Giguère[1,2], Jennifer L. Estall[5], Peter M. Siegel[1,2,6], Étienne Audet-Walsh[4*] and Arnim Pause[1,2*].

[1]Goodman Cancer Research Center, McGill University, Montréal, Québec, Canada.

[2]Department of Biochemistry, McGill University, Montréal, Québec, Canada.

[3]Canadian Centre for Computational Genomics, McGill Genome Centre, Montréal, Québec, Canada.

[4]Endocrinology - Nephrology Research Axis, Centre de recherche du CHU de Québec - Université Laval, Québec, Québec, Canada

[5]Institut de recherches cliniques de Montréal (IRCM), Montréal, Québec, Canada.

[6]Department of Medicine, McGill University, Montréal, Québec, Canada

*Corresponding Authors

Arnim Pause; arnim.pause@mcgill.ca, Phone: 1-514-398-1521; Fax: 1-514-398-6769

Étienne Audet-Walsh; etienne.audet-walsh@crchudequebec.ulaval.ca, Phone: 1-418-525-4444 x48678; Fax: 1-418-654-2298

Supplemental Figure 1

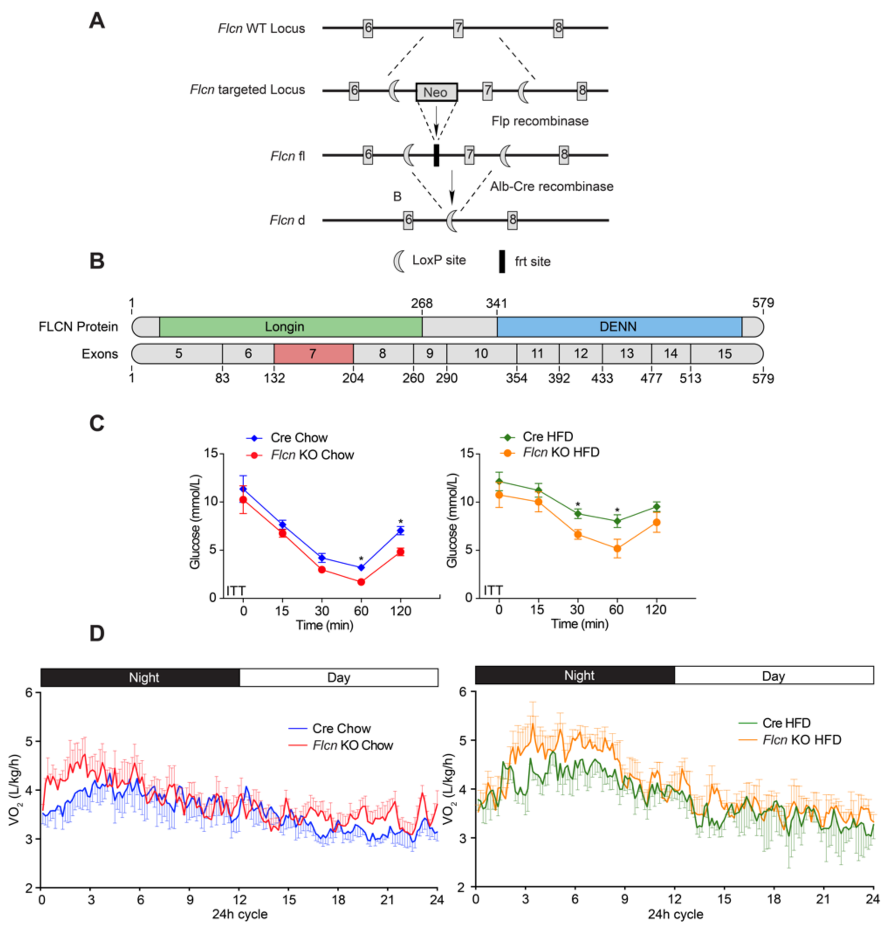

Supplemental figure 1: Generation of the liver specific *Flcn* KO model

(**A**) Schematic showing the generation of liver-specific KO mice. Mice carrying Flcn alleles flanked by loxP sites were crossed with Albumin-Cre transgenic mice to generate liver-specific Flcn knockout mice. (**B**) Schematic showing FLCN domains aligned on the corresponding exons on the mRNA sequence. Exon 7 is removed following expression of Albumin-Cre, (**C**) Blood glucose during an ITT in chow or HFD-fed mice following a 16-h fast and intraperitoneal insulin administration of 0.75 U per kg of body weight (mean ± SEM, two-way ANOVA, **P < 0.01; ***P <0.001; n = 6 mice per condition). (**D**) Metabolic cage analysis of mice fed chow or a HFD for 2 months. Circadian VO_2 consumption levels during a 12h light: 12h dark cycle.

Supplemental Figure 2

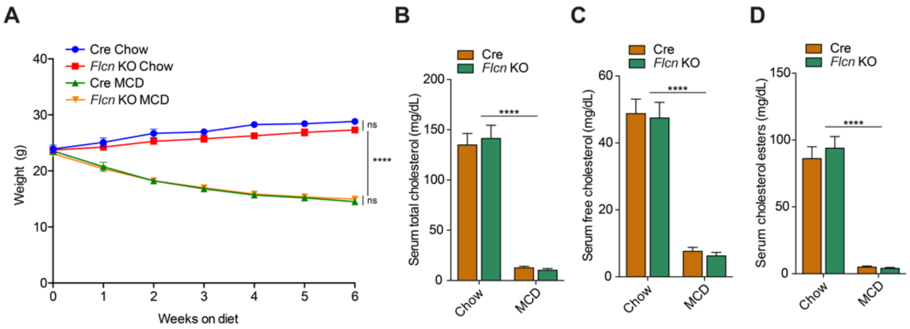

Supplemental figure 2: Weight and blood parameters are unaffected by *Flcn* loss

(**A**) Mouse body weight when fed either on chow or methionine/choline deficient diet (MCD) over a 6-week period (mean ± SEM, two-way ANOVA, ****P < 0.0001; n = 8 mice per condition). (**B**) Serum total cholesterol quantification in mice fed as described in (A) (mean ± SEM, two-way ANOVA, ****P < 0.0001; n = 8 mice per condition). (**C**) Serum free cholesterol quantification in mice fed as described in (A) (mean ± SEM, two-way ANOVA, ****P < 0.0001; n = 8 mice per condition). (**D**) Serum cholesterol esters quantification in mice fed as described in (A) (mean ± SEM, two-way ANOVA, ****P < 0.0001; n = 8 mice per condition).

Supplemental Figure 3

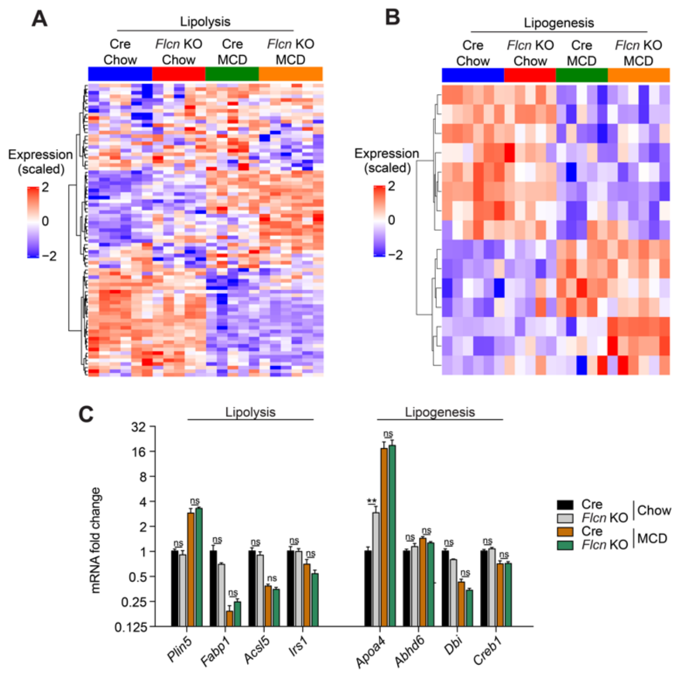

Supplemental figure 3: Gene expression analysis in liver tissues of Cre and *Flcn* KO mice

(**A-B**) Unsupervised hierarchical clustering following RNA-seq analysis of genes related to lipolysis (A, GO:0016042) and lipogenesis (B, GO:0008610) in livers of mice fed either chow or methionine/choline deficient diet (MCD) over a period of 6 weeks. (**C**) Relative quantitative real-time PCR analysis in livers of mice fed as described in (A) (mean ± SEM of the RNA fold change of indicated mRNAs; two-way ANOVA, ns = not significant; *P< 0.05; **P< 0.01; n = 8 mice per condition).

Supplemental Figure 4

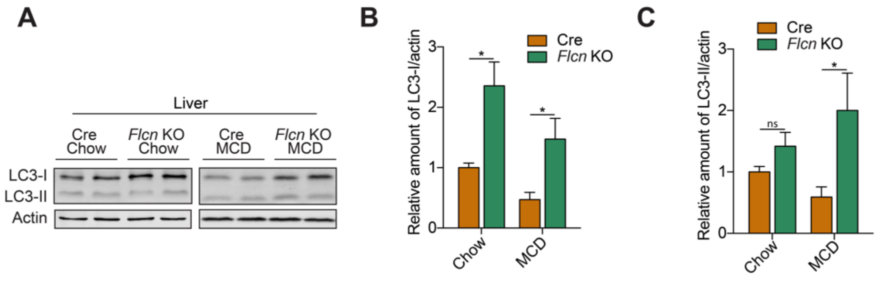

Supplemental figure 4: LC3 levels in liver tissues of Cre and *Flcn* KO mice

(**A**) Immunoblot of liver protein lysates extracted from mice fed either on chow or methionine/choline deficient diet (MCD) over a period of 6 weeks. Data are representative of 6 mice per condition. Full-length blots are presented in Supplementary Figure 5. (**B**) Quantification of the relative amount of LC3-I immunoblot. Data normalized to actin levels (mean ± SEM of the relative fold change; two-way ANOVA, *P < 0.05; n = 5 mice per condition). (**C**) Quantification of the relative amount of LC3-II immunoblot. Data normalized to actin levels (mean ± SEM of the relative fold change; two-way ANOVA, ns = non-significant, *P < 0.05; n = 5 mice per condition).

Supplemental Figure 5

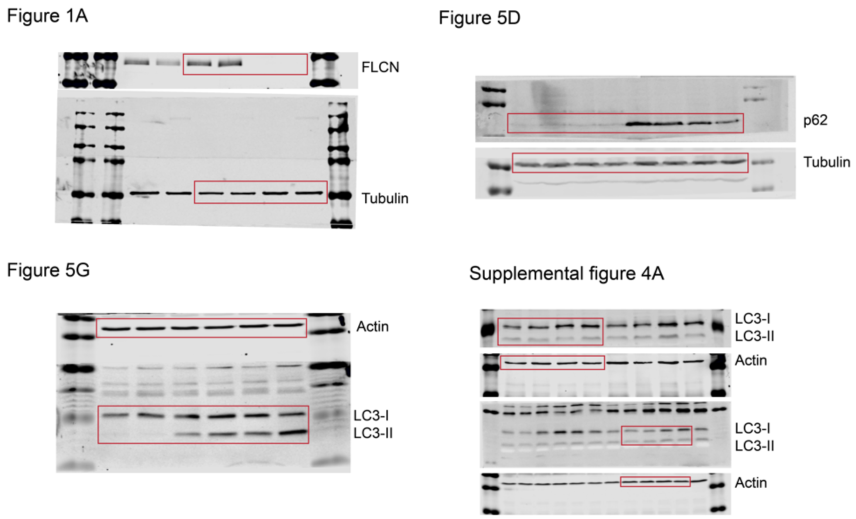

Supplemental figure 5: Full-length blots

Full-length immunoblot of the indicated figures.

Supplemental table 1: Raw RNA-seq data

QC stats, raw reads, p-values et false discovery rates following RNA-seq analysis. Total RNA from liver tissue was quantified with Illumina Nextseq500. The resulting reads were aligned to the GRCm38 mouse reference genome assembly using STAR and read counts were obtained using HTSeq. Difference in gene expression levels between the different conditions was identified with the R package limma.
